# Experience of the COVID-19 pandemic in Wuhan leads to a lasting increase in social distancing

**DOI:** 10.1038/s41598-022-23019-w

**Published:** 2022-11-02

**Authors:** Darija Barak, Edoardo Gallo, Ke Rong, Ke Tang, Wei Du

**Affiliations:** 1 Faculty of Economics, University of Cambridge, Sidgwick Avenue, Cambridge, CB3 9DD UK; 2 Institute of Economics, School of Social Sciences, Tsinghua University, Haidian District, Beijing, 100084 China; 3 School of Economics, Anhui University of Finance and Economics, Bengbu, 233030 Anhui China

**Keywords:** Human behaviour, Epidemiology

## Abstract

On 11th Jan 2020, the first COVID-19 related death was confirmed in Wuhan, Hubei. The Chinese government responded to the outbreak with a lockdown that impacted most residents of Hubei province and lasted for almost three months. At the time, the lockdown was the strictest both within China and worldwide. Using an interactive web-based experiment conducted half a year after the lockdown with participants from 11 Chinese provinces, we investigate the behavioral effects of this ‘shock’ event experienced by the population of Hubei. We find that both one’s place of residence and the strictness of lockdown measures in their province are robust predictors of individual social distancing behavior. Further, we observe that informational messages are effective at increasing compliance with social distancing throughout China, whereas fines for noncompliance work better within Hubei province relative to the rest of the country. We also report that residents of Hubei increase their propensity to social distance when exposed to social environments characterized by the presence of a superspreader, while the effect is not present outside of the province. Our results appear to be specific to the context of COVID-19 and are not explained by general differences in risk attitudes and social preferences.

## Introduction

The COVID-19 pandemic has brought the most significant and devastating global disruption since World War II with an estimated 5.5 million deaths worldwide^1–3^. Most countries implemented drastic lockdown policies to minimize infection levels, prevent healthcare systems from being overwhelmed, and reduce the number of deaths^4–7^. The first COVID-19 related lockdown started on 23rd Jan 2020 in Wuhan (Hubei) and for the subsequent 3 months the measures taken in Hubei were the strictest both within China and worldwide. In particular, according to the Oxford COVID-19 Government Response Tracker (OxCGRT), the average government response index value for Hubei in this period was 75.8 (with 0 being no measures and 100 being the maximum)^4^. Meanwhile, China as a whole scored 58.5, and the closest scoring countries—Italy and Mongolia—had an average index of 53.0 and 49.9 respectively. It is estimated that the measures implemented by the Chinese government have potentially prevented 100,000 s of COVID-19 infections^8^, and possibly contributed significantly to public health in China overall^9^.

The policies aimed at containing the spread of the pandemic have had a profound impact. Research into the impact of lockdowns and other Non-Pharmaceutical Interventions (NPIs) has documented a deterioration in physical and mental health in China^10,11^ as well as other countries^12–14^. Recent evidence shows there are mental health and burnout effects associated with the Zero-COVID policies^15^. However, to date, little attention has been paid to the effects of the pandemic on human behavior. Our paper addresses this gap.

Previous research suggests that shock events and/or drastic institutional interventions can have a long-lasting impact on behavior^16,17^. For example, colonial conscription rules in sixteenth century Bolivia and Peru led to differences in household consumption that survive to this day^18^. More recently, the 2004 tsunami in Thailand led to significant increases in prosocial behavior, risk aversion, and impatience in rural areas^19^. The sudden and drastic nature of the COVID-19 outbreak and associated lockdown policies in Hubei compared to the rest of China means they may have persistent effects on the behavior of Hubei’s residents in the short and medium term, especially when it comes to reacting to post-lockdown policies to contain the pandemic. The sudden and drastic nature of the COVID-19 outbreak and associated lockdown policies in Hubei compared to the rest of China means they may have persistent effects on the behavior of Hubei’s residents in the short and medium term, especially when it comes to reacting to post-lockdown policies to contain the pandemic.

Using an interactive web-based experiment conducted half a year after the end of the lockdown, we show that Hubei residents behave differently compared to inhabitants of other provinces in China in terms of social distancing, receptiveness to COVID-19 policies, and when exposed to a superspreader environment. In particular, we estimate that every extra 1000km between Wuhan and one’s place of residence contributes to a 7% decrease in social distancing. Using OxCGRT, we show that an increase in the harshness of lockdown measures is associated with an increase in distancing.

The differences in social distancing behavior between residents within and outside Hubei may translate into differences in social distancing policies effectiveness. We examine the effect of soft and hard policy interventions to promote social distancing. The hard policy intervention—a fine—increases social distancing only in Hubei, while the soft intervention—an informational message or “nudge”—increases social distancing both within and outside Hubei. Finally, Hubei-based participants practice more social distancing in a social environment with a superspreader. Using data from incentivized preference elicitation tasks, we find that the observed differences in behavior between Hubei residents and those from the rest of China are not explainable by general differences in preferences.

## Experimental design

### The game

**Figure 1:**
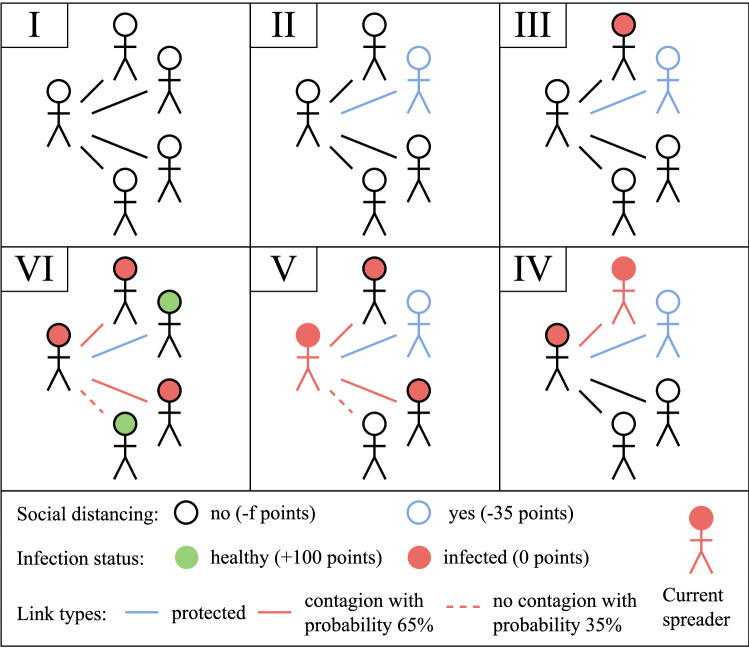
Flow of a typical round of baseline and intervention. In the experiment, we use the following parameterization: *f* = 0 points in baseline and in nudge intervention, and 15 points in fine intervention. Final payoffs for the round are a combination of individual social distancing choice and infection status. For example, a participant who practices social distancing and is healthy, receives (− 35 + 100) = 65 points. In the figure, the chosen social environment is the superspreader. In the experiment, half of the treatments had superspreader environment while the other half had a homogeneous environment.

Figure 1 presents the flow of a typical round of the experiment. Participants are randomly assigned to groups of five that stay the same throughout their involvement in the study. Within the group, in each round they are randomly assigned to five positions within the social structure—nodes on a network—as shown in Panel I. In every round each participant has to make a binary decision of whether or not to practice social distancing. Each participant has to privately decide whether to practice social distancing at a cost of 35 points. In the example in Panel II, the participant color-coded in blue is the only one who chose to practice social distancing. Once decisions are made, the computer picks one subject to be potentially infected by COVID-19 uniformly at random. If this patient zero subject practices distancing, she becomes infected with probability 50%. If patient zero does not practice distancing, infection happens for sure. COVID-19 then spreads from infected to healthy participants who do not practice social distancing with a commonly known probability of 65%. Note that those who practice social distancing cannot (a) infect others or (b) become infected through this contagious process.

An example of such contagious process is in panels III-V of Fig. 1. Panel III shows patient zero color coded in red. Given that patient zero chose not to practice distancing, there is a 65% chance that the participant in the superspreader position, who does not practice distancing either, gets infected. Panel IV shows the case when the participant in the superspreader position gets infected, and can therefore spread COVID-19 to all other participants. Finally, Panel V shows the instance when infection occurs for one out of the two remaining participants who do not practice distancing and are connected to the superspreader. Panel VI shows the final outcome of the spreading process with three participants infected and two remaining healthy.

At the end of the round, healthy participants receive 100 points while those infected get 0 points, minus costs of social distancing if applicable. For example in panel VI of Fig. 1, three participants receive a payoff of 0, one gets 65 points, and another one 100 points. Throughout both instructions and the experiment, participants are primed to think about COVID-19. For full details on the instructions and the experimental interface, consult the Supplementary Information (SI).

### Treatments

Participants play 20 rounds of the above social distancing game, which constitute the baseline part of the experiment. After these 20 rounds, they are treated with one of the policy interventions. The soft policy intervention is an informational message or *nudge*—participants must watch a 3-min video which explains how failure to practice distancing can harm others. The hard policy intervention is the introduction of a fine of 15 points for everyone who does not practice social distancing in a round of the game. Participants play another 20 rounds of the social distancing game under either the nudge or fine policy intervention. Note that the payoff structure remains unchanged in the nudge treatments (Fig. 1 with *f* = 0 points), while in the fine treatments subjects receive the fine in every round where they do not practice distancing irrespective of their health status (Fig. 1 with *f* = 15 points).

A second treatment dimension is the social environment. Participants are randomly assigned to either a *homogeneous* or a *superspreader* environment (as in Fig. 1), which stays the same throughout the 40 rounds of the experiment. In the homogeneous case, everyone is connected to everyone else in the group so an infected participant can spread COVID-19 to any other healthy participant that does not practice distancing. In the superspreader case, one participant is connected to all the others, and there are no other connections in the group. This means that any spread of infection beyond patient zero must involve the central participant either as the spreader or the recipient. A defining feature of COVID-19 is the crucial role of superspreaders in the diffusion of the disease^20,21^. For respiratory syndromes, an important determinant of being a superspreader is biological^22,23^, something that is typically unknown to the individual and outside the scope of this study. Another determinant is, however, the centrality of the individual in terms of the structure of social interactions—this is typically common knowledge and varies widely across individuals in most environments^24^. This treatment dimension, therefore, allows an investigation of how a social environment with a superspreader affects the propensity to social distance.

Using a full-factorial 2 × 2 design, we therefore obtain four treatments. As standard in the experimental literature, subjects were randomly assigned to treatments, so the effect of our treatment variables is causal. We collect at least 10 groups of 5 subjects for each of those treatments. Additionally, to investigate the impact of Hubei residence on behavior, we ran these four treatments separately in Hubei and rest of China. We summarize the details of our dataset next.

### Dataset

**Figure 2:**
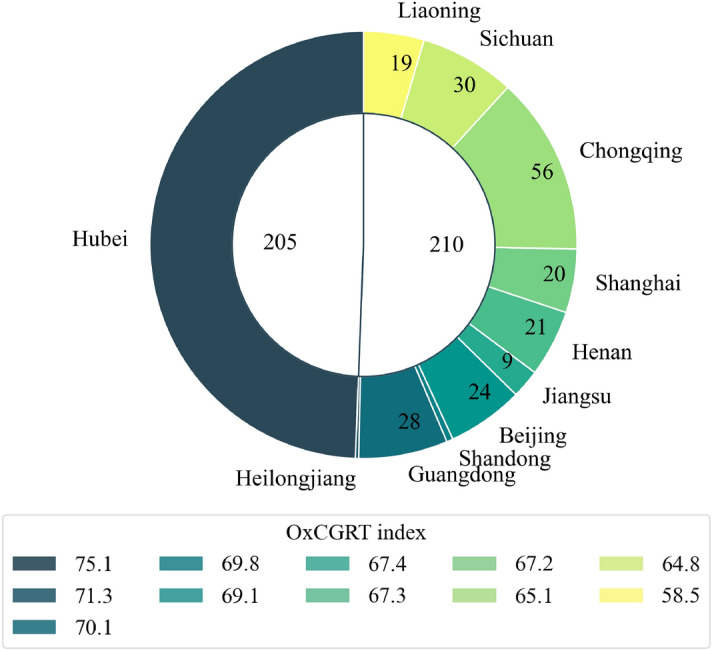
Distribution of subjects from different Chinese provinces. Color coding indicates the average values of the OxCGRT index for the provinces.

Using a local recruitment company, we sourced 415 participants from 11 Chinese provinces. Figure 2 displays the proportion of participants from each province. In the final sample, 205 subjects (41 groups) are from Hubei province and 210 (42 groups) from the rest of China. We verify the place of residence using (1) self-reported data from the recruitment survey, (2) data from the survey company, and (3) IPs of subjects collected when completing the experiment. Despite the possibility of selection bias, we obtain a diverse sample in terms of age and gender. In particular, 47.3% of our sample is female and the mean age is 35 years (s.d. 10 years). Figure 2 also shows the average stringency of lockdown measures in the 11 provinces in our sample over the period of the Hubei lockdown as measured by the 0–100 scale of the OxCGTR index. Note that throughout the paper we focus on the difference between Hubei and other 10 provinces which were under more moderate lockdown measures. Further note that since we have between 1 and 56 subjects from each of the other 10 provinces, we cannot comment on the differences between these provinces.

## Results

We analyze the data using a linear probability model, where the individual decision to practice social distancing is the dependent variable (binary), and the controls are (1) our treatments, (2) a set of demographic variables, and variables for personal preferences, plus (3) a variable capturing the distance of one’s place of residence from Wuhan. Estimated coefficients from this model are in M1 Table 1.

**Table 1:** Main regression results for individual propensity to social distance.

Dependent variable:	Social distancing (binary)					
Model:	M1		M2		M3	
**Independent variables**						
Fine treatment	0.0341*	(0.0195)	0.0343*	(0.0195)	0.0343*	(0.0195)
Nudge treatment	0.0605***	(0.0148)	0.0603***	(0.0148)	0.0603***	(0.0148)
Superspreader environment	− 0.0491	(0.0314)	− 0.0494	(0.0312)	− 0.0481	(0.0311)
Distance from Wuhan (100's km)	− 0.0070**	(0.0031)				
Hubei residence (1 = yes)			0.0852***	(0.0311)		
OxCGRT index					0.0076**	(0.0034)
Constant	0.192	(0.2080)	0.101	(0.2110)	− 0.389	(0.3660)
No of observations	8280		8280		8280	
No of subjects	414		414		414	

Standard errors (reported in parentheses) are clustered at the group level. Significance level: ***p < 0.01, **p < 0.05, *p < 0.1. (a) All regressions use Linear Probability Model. (b) All models include the following controls: gender dummy (female = 1), age, years of education, employed or entrepreneur dummy (yes = 1), religious dummy (yes = 1), risk score (as captured by BRET), prosocial values (as captured by SVO; yes = 1). (c) To account for learning effects, we discard the first 10 rounds of baseline and intervention, and only consider 20 remaining rounds per subject. See Materials and Methods for details. (d) One subject did not complete the post experimental questionnaire and BRET.

### Hubei province versus the rest of China

Our first finding is that the individual propensity to practice social distancing in our experiment is inversely related to distance from Wuhan to one’s place of residence. We estimate that every extra 1000km between Wuhan and one’s place of residence contributes a 7 percentage point decrease in the probability of social distancing. In practical terms, this suggests that the individual propensity to do social distancing of residents of Chongqing, which is approximately 723km away from Wuhan, to be 5 percentage points less than that of Wuhan residents. The effect is statistically significant in all our specifications and robust (M1, *p* = 0.02). Replacing the distance variable with a dummy equal to one for Hubei subjects (M2 Table 1), we estimate that the probability of social distancing is 8.5 percentage points higher in Hubei province than outside of it (M2, *p* = 0.006).

We hypothesize that distance from Wuhan captures heterogeneity in the harshness of the lockdown policy experienced by people from different parts of China. While tight COVID-19 related restrictions were generally experienced throughout the world in early 2020, Wuhan was the first to go under total lockdown for a nearly 3-months period together with its 11 million residents^25^. According to the OxCGRT, Hubei province has spent the whole 23rd Jan–2nd May 2020 period in a very strict lockdown, whereas other provinces (with the exception of Heilongjiang) mostly experienced more moderate measures^4^. To test this hypothesis, we use data from the OxCGRT, which tracks harshness of government response to the COVID-19 pandemic globally. We focus on the 23rd Jan–2nd May 2020 period, and calculate the average of the overall government response index for each of the provinces in our sample. In this way we obtain a single index on a [0, 100] scale. The correlation between distance from Wuhan and this index for the 20 cities in our sample is − 0.6683 (*t* test, *p* = 0.001). Replacing distance of one’s place of residence from Wuhan by the index, we estimate that a 1 point increase in the response index corresponds to a 0.75 percentage point increase in individual propensity to do social distancing in our experiment, which is significant in all specifications considered (M3 Table 1, *p* = 0.03).

An important caveat is that the lockdown measures were not imposed randomly—stricter measures were put in place in provinces with more severe COVID-19 outbreaks. The Hubei outbreak was by far the largest in China, with over 68,300 infections and about 4500 COVID-19 related deaths confirmed at the time of writing^26^. Guangdong—the second province by the size of the outbreak—recorded approximately 3300 cases and 8 deaths. Indeed, the correlation between measures of harshness and number of confirmed cases for 11 provinces in our sample is 0.6835 (*t* test, *p* = 0.001). An alternative interpretation of our results is, therefore, that the experience of the severity of the outbreak, rather than the lockdown measures, is the primary driver of the behavioral difference. While we are unable to differentiate between these alternative channels, the central message remains that what Hubei participants experienced caused a lasting change in their social distancing behavior.

The association between social distancing behavior and the severity of the COVID-19 pandemic experience raises two natural questions. The first one is whether there is a difference between participants from Hubei, who lived through a larger outbreak and harsher lockdown, and those from the rest of China in terms of their behavioral responses to our treatment variables—fine/nudge policy interventions and homogeneous/superspreader social environments. The second one is whether the association is driven by specific demographic characteristics and/or risk/social preferences. In order to investigate this, we repeat our core analysis but interact every variable with a dummy equal to one if a subject is from the Hubei province and zero otherwise. This is equivalent to fitting the model separately on the two datasets. The results are reported below, and the full table with coefficients can be found in the SI.

### Response to intervention

**Figure 3:**
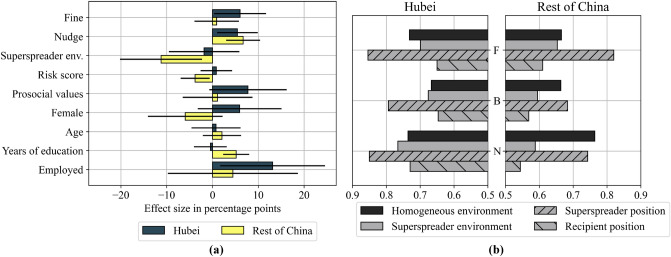
(**a**) Estimated effect size on the probability of social distancing for treatment variables and selected controls, split by Hubei and rest of China. Note that: (1) risk score estimates are reported for an extra 25 points, (2) Prosocial = 1 for subjects with prosocial values as classified by the SVO scale, (3) Female = 1 for female subjects, (4) age estimates are reported for an extra 10 years, (5) Employed = 1 if subject is either employed or runs their own business. (**b**) Probability of doing social distancing separately for Hubei and rest of China in baseline part of the experiment (B), and under fine (F) and nudge (N) interventions split by social environment and positions in the superspreader environment.

The hard policy intervention of introducing a fine increases the propensity to social distance in Hubei, but not in the rest of China. As shown in Fig. 3a, the fine leads to a significant 6.0 percentage points increase in individual propensity to social distance in Hubei (*t* test, *p* = 0.04), while outside of Hubei the estimated effect is only 0.9 percentage point and not significant (*t* test, *p* = 0.7). The difference between the two effects is not statistically significant (*t* test*, p* = 0.2).

In contrast, the soft policy intervention (the nudge) increases the propensity to social distance throughout China. As shown in Fig. 3a, the estimated size of the effect is 5.4 percentage points (*t* test, *p* = 0.02) in Hubei province and 6.7 percentage points (*t* test, *p* = 0.0004) in the rest of China. The nudge is marginally more effective than the fine in the rest of China (*t* test, *p* = 0.06), while the effectiveness of the two policy interventions is indistinguishable in Hubei (*t* test, *p* = 0.9). Note that the difference in the effectiveness of the nudge in the Hubei province and outside of it is not statistically significant (*t* test, *p* = 0.7).

### Response to social environment

A theoretical analysis of the social distancing game assuming self-interested rational individuals predicts that the individual propensity to do social distancing should be higher in the homogenous social environment. This stems from the fact that the density of connections is higher than in the superspreader case (see SI). This may, however, differ behaviorally because the diffusion of COVID-19, especially on the outset, was driven by superspreading events^20,27,28^. Figure 3a shows that the theoretical prediction is validated in the rest of China—participants’ propensity to do distancing is 11.1 percentage points higher in the homogeneous compared to the superspreader environment (*t* test, *p* = 0.02). In contrast, participants from Hubei do as much distancing in the superspreader environment as they do in the homogeneous one—the difference is only 1.8 percentage points and it is insignificant (*t* test, *p* = 0.5).

Figure 3b delves deeper into the behavior in the superspreader environment between Hubei and rest of China participants. It reports average propensity to practice distancing split by place of residence, type of intervention, and position in the social environment. The left panel shows that Hubei-based participants in the superspreader position do significantly more social distancing relative to those in the homogeneous environment, while recipients do about as much as those in the homogeneous social structure. This is true for both the baseline part of the experiment (middle set of bars), and the two interventions (top and bottom sets of bars). The right panel shows that the behavior of the participants in the rest of China is quite different. Here, subjects in the superspreader position do as much social distancing as in the homogeneous environment particularly in the baseline part of the experiment and following a nudge intervention. In contrast, the recipients perform significantly less distancing relative to both the superspreader and the homogeneous environment throughout the experiment.

To confirm these observations, we repeat our core analysis, but instead of using a single dummy for a superspreader environment, we include one for each of the types of positions in this environment (see the SI for the full table with the coefficients). The results of this exercise confirm our observations. In Hubei the superspreader has a 8.9 percentage points higher propensity to social distance compared to participants in the homogeneous environment (*t* test, *p* = 0.02), and peripheral participants do as much distancing as participants in the homogeneous environment (*t* test, *p* = 0.3). In contrast, in the rest of China, superspreader participants do as much distancing as participants in a homogeneous environment (*t* test, *p* = 0.6), and peripheral participants have a 14.7 percentage points lower propensity to social distance compared to the ones in a homogeneous environment (*t* test, *p* = 0.002).

### Risk aversion and social preferences

An alternative explanation for our findings on the behavioral differences between participants from Hubei and the rest of China is that the two subject pools differ in terms of their general attitudes toward risky behavior. In fact, past research shows that natural disasters can sometimes lead to persistent increases in risk aversion^19^. As part of the experiment, we collect subjects’ attitudes to risk using an incentive-compatible ‘Bomb’ risk elicitation task (BRET)^29^. The task amounts to deciding how many boxes to collect from a maximum of 100, with more boxes translating into potentially higher earnings, but also a higher risk of collecting a (hidden) bomb that destroys all earnings. Theory predicts that a risk-neutral subject collects 50 boxes with lower values indicating greater risk aversion. The average subject in our sample is moderately risk-averse with a BRET score of 42, which is consistent with previous findings in the literature^29^. Additionally, as part of recruitment, we collect subjects’ self-reported attitudes to risk^30^.

There is no difference in general risk attitudes between the Hubei and rest of China participants according to either the BRET score (Mann–Whitney test, *p* = 1.0) or the self-reported risk index (Mann–Whitney test, *p* = 0.2). In other words, the differences between Hubei and rest of China participants seem to be confined to behaviors related to the COVID-19 pandemic, i.e. social distancing, rather than general behavior. Interestingly, Fig. 3a shows that the propensity to do social distancing is increasing with risk aversion (as captured by the BRET score) for participants in the rest of China (*t* test, *p* = 0.02), but there is no significant association for Hubei-resident participants (*t* test, *p* = 0.6). A potential explanation is that the harsh experience of the lockdown and/or pandemic in Hubei generates a widespread attitude toward social distancing that is independent of generalized risk preferences, while in the rest of China the propensity to practice social distancing is, as one would expect, increasing with risk aversion.

A second alternative explanation for our findings is that participants from Hubei have a less self-interested attitude compared to participants from other parts of China, and therefore they practice more social distancing to benefit others. The study of the effects of the 2004 tsunami in Thailand suggests that ‘shock’ events may lead to more prosocial behavior^19^. We collect subjects’ social preferences using an incentivized 6-item Social Value Orientation (SVO) task^31^. The underlying idea of the SVO framework is that people vary in terms of their motivations when evaluating different allocations of resources between themselves and others. In our sample, 51% of subjects are individualists and 49% are prosocials, and therefore we use a binary variable to capture social preferences.

There is no difference in social preferences between participants from Hubei and the rest of China according to the SVO score (Mann–Whitney test, *p* = 0.5). This indicates that the observed differences in behavior by Hubei participants are specific to social distancing attitudes, rather than general differences in preferences. In general, we would expect that participants with prosocial values are more likely to practice social distancing in the experiment compared to individualists because social distancing benefits others in their group. Figure 3a shows that subjects from Hubei province with prosocial values are marginally more likely to do social distancing with an average difference of 7.7 percentage points (*t* test, p = 0.07), while the difference is only 0.6 percentage points in the rest of China and is not significant (*t* test, *p* = 0.9).

### Demographic characteristics

When it comes to demographic characteristics, we find two heterogeneities between Hubei province and the rest of China (Fig. 3a). First, while more education is associated with significantly more distancing outside of Hubei province, the effect is not present within Hubei. In particular, an extra year of education is associated with 5.2 percentage points more distancing in the rest of China (*t* test, *p* = 0.0005), while in Hubei the estimated size of the effect is 0.5 percentage point and is insignificant (*t* test, *p* = 0.8). Second, subjects from Hubei province who were either employed or owned a business at the time of conducting the experiment did significantly more social distancing in the experiment, but the same is not true outside of it. The estimated effect in Hubei is 13.1 percentage points (*t* test, *p* = 0.02), and 4.4 percentage points in the rest of China (*t* test, *p* = 0.5). Note that the effects of age and gender are not significant in our experiment. This may be partly explained by the fact that our sample is restricted to adults. In particular, existing research into adolescents suggests that younger people may differ in their attitudes to lockdowns and social distancing relative to an adult population^32^.

## Discussion

The sudden outbreak of COVID-19 in Wuhan in early 2020 demanded a quick and decisive response from the government. To limit the spread of the virus, and potentially save tens of thousands of lives, the Chinese government implemented a very strict lockdown which affected most of Hubei province and lasted almost three months. Our experiment is the first to shed light on the possible medium-/long-term effects brought by the outbreak and the associated lockdown.

Our first result is that the level of social distancing is significantly higher in our experiment in Hubei province than outside of it. Both the distance of one’s place of residence from the epicenter of the outbreak—Wuhan—and the strictness of government response to COVID-19 during the initial lockdown are robust predictors of individual propensity to social distance. Our experiment does not attempt to discriminate between the two plausible explanations—severity of the outbreak and strictness of lockdown—but it is clear that the overall experience of Hubei residents has created a behavioral difference that is persistent half a year after the end of the outbreak.

Our second result is that a soft intervention in the form of an informational video (a nudge), which highlights the harm caused to others by not practicing social distancing, is effective at increasing individual propensity to social distance throughout China. In contrast, a hard intervention in the form of a fine for non-compliance seems to work in Hubei province but not outside of it.

Our third result is that subjects in Hubei and the rest of China react differently to a superspreader type of social environment. Participants in the rest of China largely respond in line with theoretical predictions based on a standard game-theoretic framework, while participants from Hubei province violate these predictions. In particular, in a superspreader environment the recipients do not decrease their social distancing relative to the homogeneous environment, while superspreaders increase their propensity to social distance. This leads to a higher level of social distancing overall.

We find that the above behavioral heterogeneities cannot be explained by standard measures of general risk aversion or social preferences. The data suggests that these differences in behavior are specific to the context of social distancing and COVID-19, rather than general differences in preferences between Hubei residents and those from the rest of the country. Note that our list of controls is non-exhaustive, and other potential explanations may contribute to the observed differences in behaviour. For example, it has been shown that in China people with anxiety and depression are more willing to pay for a COVID-19 vaccine^33^.

Finally, our study highlights the important role that interactive web-based experiments can play in investigating people’s behavior, and how behavior can be affected by ‘shock’ events. Even though the decision situation faced by subjects in our experiment is artificial, we find clear and robust differences in behavior of subjects from the Hubei province and the rest of China, even after controlling for demographic characteristics and social preferences.

## Methods

Full details on methods, including theoretical framework, data collection and data analysis methods, together with a detailed description of the dataset are in the Supplementary Information (SI).

### Ethical approval

This research received ethical approval for the use of human subjects from the Faculty of Economics Ethical Committee (University of Cambridge, ref.UCAM-FoE-20-02) and the Department of Psychology Ethics Committee (Tsinghua University, ref.THU202019). The experiment was performed in accordance with the relevant guidelines and regulations. Informed consent was obtained from all subjects before participation.

### Software

The experiment was coded in oTree (v2.2.4)^34^ with a server hosted on Heroku (http://www.heroku.com).

### Recruitment and sessions

Subjects were recruited using the local survey company Wenjuan which is affiliated with Zhongyan Technology. During recruitment, we collected information on basic demographics, including gender, age, and place of residence. The experiment was conducted between October 3rd and November 14th 2020, and involved a total of 30 sessions with 1–5 groups each. The experiment took an average of 59 min (s.d. 20 min) to complete, and subjects earned an average of 17.7 yuan (s.d. 3.5 yuan). Subjects remained anonymous throughout both recruitment and experiment, and repeated participation was not allowed.

### Learning effects

We identify significant learning effects in the early rounds of baseline and intervention parts of the experiment. In effect, subjects tend to converge to a particular stable strategy (e.g. always practice social distancing) after several rounds of the experiment. Therefore, in the analysis we use the last 10 rounds of the baseline and intervention parts of the experiment, but all results are robust to using all data. Full details on convergence analysis and relevant robustness checks are in the SI.

### Statistical analysis

Since our dependent variable is binary, our analysis relies on a Linear Probability Model^35,36^, but the estimates are robust to using a Logit or a Probit model instead (see SI).

## Supplementary Information

Supplementary Information.

## Data Availability

The dataset collected and analyzed during the current study is available from the corresponding author (Edoardo Gallo, email: edo@econ.cam.ac.uk) on reasonable request.
