# Supplementary material for: Experience of the COVID-19 pandemic in Wuhan leads to a lasting increase in social distancing
Source: Sci Rep. 2022 Nov 2;12:18457. doi: 10.1038/s41598-022-23019-w (PMC9628316; doi:10.1038/s41598-022-23019-w)
Supplement: Supplementary file 1 — Supplementary Information. [file 41598_2022_23019_MOESM1_ESM.pdf]

Supplementary Information to "Experience of the COVID-19 pandemic in Wuhan leads to a lasting increase in social distancing"

Darija Barak, Edoardo Gallo, Ke Rong, Ke Tang, Wei Du

October 5, 2022

This Appendix contains supplementary information. Section 1 specifies our theoretical framework, calibration for the experiment, and the set of hypotheses we investigate. Section 2 describes our methodology of data collection, including recruitment and the actual experiment. Section 3 presents our dataset, and Section 4 explains our data analysis methodology. Section 5 presents our key results, while Section 6 summarizes our key robustness checks. Finally, Section 7 presents English translations of original instructions written in Mandarin.

1 Theory and hypotheses

In this section we present the theoretical model underpinning our experimental design. We also present our calibration of the model for the experiment, and list the key hypotheses that we test in Section 5.

The model. A set of $N = 1, 2, \dots, n$ of risk-neutral agents are located on an unweighted and undirected network $\mathbf{G}$. $G_{ij} = G_{ji} = 1$ when there is a link between agents i and j, else $G_{ij} = G_{ji} = 0$. Agents simultaneously decide whether to practice social distancing, at a private cost $c > 0$.

One agent is then chosen to be exposed to COVID-19 uniformly at random. In what follows, we refer to her as patient zero. If partient zero practices social distancing, she becomes infected with COVID-19 with probability $\gamma < 1$, otherwise, she is infected for sure. Any infected agent who is not practicing social distancing, including patient zero, can pass COVID-19 through contagion to her healthy neighbors who are not practicing social distancing with probability $\alpha \in [0, 1]$. Any agent who practices social distancing cannot pass the disease to others through contagion, or get infected through contagion

herself.

Once contagion is over, payoffs are calculated. A healthy agent receives a benefit $b > c$, while an infected agent earns 0 benefit. Additionally, any agent who decided to practice social distancing pays a cost $c > 0$ regardless of her infection status.

Define the subset of agents who practice social distancing $S \subseteq N$, and the probability that an individual agent i is infected $p_{i|S_i}$. Observe that if $i \in S$ then $p_{i|S_i} = \gamma/n$, since i can only become infected if she is patient zero. Assuming a self-interested risk-neutral agent framework, the expected payoff to agent i depends on her actions and the subset of agents who practice distancing. Specifically:

$$\pi_i = \begin{cases} (1-\gamma/n)b - c, & \text{if } i \in S \\ (1-p_{i|S_i})b, & \text{otherwise.} \end{cases} \tag{1}$$

We use the following concepts in our analysis – the socially optimal subset of agents who are practicing distancing, and the pure strategy Nash equilibrium subset. We define the socially optimal set subset of agents practicing distancing as one that maximizes the total expected payoff of the group. In cases where the expected payoff of agent i is unchanged regardless of whether of not she is in the subset, we assume that she is. The pure strategy Nash equilibrium subset of agents who practice social distancing is such that every agent who belongs to the subset weakly prefers to be in the subset, whereas any agent not in the subset prefers to be outside of the subset.

As discussed below, for some parameterizations of the model, the socially optimal subset of agents practicing distancing and the Nash equilibrium subset need not coincide. This then creates inefficiencies in the form of under- or over-provision of social distancing which is effectively a public good [1]. One way to correct the inefficiency is by introducing a fine for not practicing social distancing $f > 0$. The fine then adjusts the relative attractiveness of practicing social distancing by modifying expected payoff of agent i such that:

$$\pi_i = \begin{cases} (1-\gamma/n)b - c, & \text{if } i \in S \\ (1-p_{i|S_i})b - f, & \text{otherwise.} \end{cases} \tag{2}$$

Note that since the fine increases the costs of not practicing social distancing, we should expect it to weakly increase the amount of social distancing in a network regardless of its architecture.

Parameterization. To narrow down the scope of our theoretical analysis to results that are relevant for this experiment, we fix some parameters of the model as follows. First, we focus on setups with five agents – i.e. $n = 5$. Next, we consider two network architectures. One is a complete network, where all nodes are connected to each other, the other is a star network where the central node – the hub – is connected to all other nodes, and no further links are present. To fix ideas, in what follows, we refer to these networks as homogeneous and superspreader environments. We refer to nodes in the homogeneous environment as 'H', while those in the superspreader environment are 'S' for the superspreader, and 'R' – for the recipient. Further, we set the (1) cost of practicing social distancing $c = 35$ points, (2) the benefit from being healthy $b = 100$ points, (3) the probability that patient zero who practices distancing is infected $\gamma = 0.5$, and (4) the probability with which an infected agent can pass COVID-19 to other agents not practicing distancing $\alpha = 0.65$. Finally, in some of our treatments we set the fine for not practicing social distancing $f = 15$ points (and 0 in other treatments). In other treatments, we replace the fine by a nudge – i.e. a 3-minute video which highlights the harm to others of not practicing distancing.

Consequently, we have a 2×2 full factorial design with two social environments – homogeneous and superspreader, – and two types of intervention – fine and nudge. Note that all subjects in the experiment first play the game without an intervention, and then are subjected to one of the two interventions.

Theoretical results. Having set out the parameters, we can derive theoretical predictions of the model. Full set of proofs for our hypotheses is available from the authors on request.

Hypothesis 1 *The average propensity to do social distancing is higher in the homogeneous environment compared to the superspreader.*

Given our parameterization, theoretical analysis of the above model predicts that in a pure strategy Nash equilibrium in a homogeneous environment 3 agents should practice social distancing. Note that since all agents are identical, there is no unique pure strategy Nash equilibrium subset. On the other hand, for the superspreader environment, the unique Nash equilibrium is such that only the superspreader practices distancing but not the recipients. It follows, that the average individual uptake of social distancing in the homogeneous environment is 0.6, whereas in the superspreader environment it is 0.2.

Hypothesis 2 *There is under-provision of social distancing in the homogeneous environment relative to the social optimal but not in the superspreader environment.*

Theoretical analysis of the above model suggests that the socially optimal subset of agents who practice social distancing is any 4 of the 5 agents. Given that the Nash equilibrium involves 3 agents practicing social distancing, the model predicts an under-provision of social distancing. Conversely, in the superspreader environment, the socially optimal subset of agents who practice distancing coincides with the Nash equilibrium subset – i.e. only the superspreader practices distancing. Therefore, there should be no under- or over-provision of distancing in the superspreader environment.

Hypothesis 3 *A fine* $f = 15$ *points for not practicing social distancing increases the amount of social distancing.*

We expect the fine to increase the amount of social distancing both in the homogeneous and superspreader environments. Note that the calibrated size of the fine is such that it should correct the inefficiency in the homogeneous environment, and have no effect or actually create an over-provision of social distancing in the superspreader environment. While the fine alters expected payoffs by making not practicing distancing more costly, the nudge highlighting the harm to others of not practicing distancing has no impact on the expected payoff of a self-interested rational agent. We therefore expect the nudge to have no effect on behavior regardless of the environment.

Hypothesis 4 *A nudge highlighting the harm to others of not practicing social distancing has no effect on agents' social distancing decisions.*

Consequently, we expect the fine to be more effective than the nudge.

Hypothesis 5 *A fine* $f = 15$ *points increases the amount of social distancing weakly more than the nudge.*

Finally, we hypothesize that real-world experience of a severe outbreak of COVID-19 may have an effect on behavior. Existing literature identifies behavioral effects of interventions, and generally 'shock' events that persist well beyond the actual duration of the policies themselves [2, 3]. We therefore think that the Hubei experience of the COVID-19 pandemic together including the strict lockdown that suddenly severely limited movement of 11 million citizens of Wuhan may have persistent effects on behavior of Hubei's residents, even after the lockdown was lifted on May 2nd 2020. In particular, we

hypothesize that Hubei resident may exhibit greater risk aversion when it comes to contagious environments, and so practice more social distancing in our stylized game. We formulate the following research question.

Research Question 1 *Does the experience of the outbreak of the COVID-19 pandemic and a total lockdown in Hubei province increase the amount of social distancing?*

To investigate this research question, we sample roughly half of our subject pool from the Hubei province, and the other half – broadly from the rest of China. Further details are in Section 3.

2 Methodology: data collection

In this section we describe our data collection procedures. Section 2.1 explains how we recruited subjects. Section 2.2 and Section 2.3 explain the workflow and implementation of the experiment.

2.1 Recruitment

We recruited subjects using Wenjuan – a local recruitment company affiliated with Zhongyan Technology (see `https://www.wenjuan.com/`). Subjects were sourced from 20 cities across 11 provinces to give us a diverse sample that is broadly representative of the urban population of China in terms of their geographical location and gender. Potential subjects were asked to fill in a quick survey and complete a qualifier quiz.

The recruitment survey takes an average of 5 minutes to complete and pays a fixed reward of 5 yuan. As part of the survey, we collect information about participants' age, gender, province and city of residence, experience with decision-making experiments, and self-reported attitudes to risk [4]. Additionally, we inform participants about the upcoming interactive experiment and collect their consent for participation. Further, subjects are asked 2 (out of 4 randomly chosen) questions aimed at testing basic understanding of probability theory. Participants are given 3 attempts at the questions and must answer both correctly.

Participants who correctly answer the qualifying questions take part in a bonus task for a chance to win an amount in the 1.5-10 yuan range. The bonus task is the 6-item Social Value Orientation (SVO) task [5]. The task is aimed at eliciting subjects' social preferences, and classifies subjects as belonging to one of the four categories – individualistic, competitive, prosocial, and altruistic. In practice, for each of the 6 decisions of the SVO task, participants are asked to choose between 9 different allocations of

money between themselves and another anonymous person. These preferences are then used to determine subjects' types. For every 50 participants who completed the bonus task, we randomly selected 2 and implemented one randomly drawn decision of theirs. The instructions for the SVO scale from the recruitment survey are in Section 7.

2.2 Experiment

Subject receives a link to our portal at the time advertised to her in an invitation. To join the session, she needs to click on the link and authenticate on our portal using a random ID issued to her during recruitment. As soon as she authenticates, she starts working through the instructions for the first part of the experiment (henceforth, Baseline). Instructions are followed by an understanding quiz, which the participant must pass to qualify for the experiment. The quiz has 3 question, and the participant must answer all of them correctly. Subject has 3 attempts at the quiz, and every unsuccessful attempt is followed by an explanation of correct answers. The instructions and the quiz take an average of 8.3 minutes to complete (s.d. 5.2 minutes). Throughout the instructions, subject is primed to think about COVID-19. Full instructions together with the quiz are in Section 7. After passing the quiz, the participant joins a waiting room where she waits to be matched in a group with other subjects. Subject is compensated for waiting at a rate of 0.2 yuan for every 20 seconds of waiting up to a maximum of 5 yuan. Waiting time is capped at 10 minutes, and if the subject is not allocated into a group within 10 minutes, she receives compensation for reading the instructions, passing the quiz and waiting. Group allocation is randomized. Once a group is formed, participants proceed to Baseline where they play 20 rounds of the same game.

Each round of the game begins with subjects being randomly allocated to five positions in the environment – either homogenous or superspreader – which is constant throughout the experiment. Subjects learn their position in the environment and are asked to privately make their social distancing decisions at a cost $c = 35$ points. In each round, a subject has 80 seconds to make their decisions, otherwise she receives a penalty of 50 points. Failure to submit decisions in three consecutive rounds results in disqualification from the experiment without compensation.

Once decisions are made, one subject is randomly selected to be patient zero. If patient zero practices distancing, she becomes infected with COVID-19 with probability 50%, else she is infected for certain. If patient zero does not practice distancing, COVID-19 then spreads through the environment through contagion to other participants who chose not to practice distancing uniformly at random with probability 65%. Note that those who practice distancing, cannot infect others or themselves become

infected through contagion.

Once the contagious process is over, payoffs for the round are calculated. Healthy subjects earn 100 points, while those infected earn zero. Further, all subjects who decided to practice distancing pay 35 points regardless of their infection status. At the end of each round, subjects learn their outcomes for the round, and can also see their history of play for the last 5 rounds. Subjects have 20 seconds to review this information. Note that subjects are not informed of the identity and/or decisions and outcomes of other members of their group at any point during or after the experiment. This is done to suppress effects of others' decisions and outcomes on individual choice.

After the Baseline part of the experiment, participants proceed to the instructions for the Intervention, which depend on the treatment. In the fine treatment, subjects are explained that, in the next part of the experiment, they will receive a fine of $f = 15$ points every time they do not practice social distancing regardless of their infection status. In the nudge treatment, subjects must watch a 3-minute video which highlights the costs to others of not practicing social distancing. Instructions for the intervention take on average 2.7 minutes (s.d. 1.7 minutes). Note that the nudge instructions take on average twice as long as the fine due to the need to watch a video. Further details and instructions for the two types of intervention are in Section 7. In both cases, the instructions are followed by a 1-question understanding quiz, asking subjects to identify the difference between Baseline and Intervention. The quiz serves essentially as an attention check, and participants have 3 attempts to answer the question. Once all members of a group pass the quiz, they proceed to Intervention.

In Intervention, participants play further 20 rounds of the same game. The group composition and the social environment remain the same as in the Baseline. Further, for fine treatments, subjects receive a fine $f = 15$ points in every round where they decided not to practice distancing. For treatments with the nudge, the payment structure remains unchanged from the Baseline.

Once subjects complete Intervention, the interactive part of the experiment is over. Subjects then proceed to the Post-experimental Questionnaire. Here, we ask a set of standard demographics questions. We repeat some of the questions from recruitment survey – i.e. about gender and age – to check answers for consistency.

Finally, a subject completes a Bomb Risk Elicitation Task (BRET) to elicit risk preferences [6]. Subject is presented with 100 boxes arranged on a 10×10 matrix. One of these boxes contains a bomb but the location of the bomb is unknown. She is asked to choose how many boxes she want to collect. Boxes are collected from the top-right corner of the matrix, left to right, at a rate of one box per second. Participant must decide when to stop collecting boxes. After the boxes are collected,

the contents of the boxes are revealed. If the bomb is collected, it explodes and reduces participant's earnings for BRET to zero. Otherwise, the participant earns 0.1 yuan for each collected box. Assuming a power utility function, a risk-neutral participant opens 50 boxes. If a participant opens more than 50 boxes she is considered risk-seeking, and if she opens fewer than 50 boxes, she is considered risk-averse. Instructions for the BRET are in Section 7.

Upon completing BRET, subject is taken to the Payment Page, where she can see her earnings for the experiment. She can also scroll through her history of play in Baseline and Intervention. All participants receive a fixed fee of 5 yuan. Additionally, subjects earn a bonus for the interactive part of the experiment, BRET, and any waiting time if applicable. To reduce wealth effects [7], for the interactive part of the experiment subjects are paid for 4 randomly chosen rounds of Baseline and Intervention. Earnings are converted at a rate of 50 points per 1 yuan.

On average, the experiment takes 59 minutes (s.d. 20 minutes) to complete and pays 17.7 yuan (s.d. 3.5 yuan) including a 5 yuan fixed fee. Upon completing the experiment, participants receive a code to submit to the survey company who then process payments.

2.3 Implementation

We conducted the experiment between October 3rd and November 14th 2020. The main experiment was programmed in o-Tree (v2.2.4; `www.otree.org`) [8] with a server deployed on Heroku (`www.heroku.com`). For the BRET task, we used a modified version of the implementation by Holzmeister and Pfurtschelle [9].

We collected the data in a total of 30 sessions, each with 1-5 groups. Session assignment to treatment was randomized. A typical session had 25-60 places which were allocated on the first-come-first served basis. We accepted new subjects for 10 minutes since the start of the session, or until no more spaces were available.

Only those participants who correctly answer qualifying questions in the recruitment survey and give their consent for participation in the experiment were invited to the main experiment. Further, we kept track of IP addresses of subjects who have already completed the experiment, and excluded those with duplicate IP addresses from the list of invitees. Finally, as explained in Section 2.2, subjects must pass two understanding quizzes during the main experiment to qualify for participation.

When running pilots for this experiment, we discovered that a small proportion (4%) of subjects suffered from random latency issues. Consequently, even though they were able to join the experiment and get allocated to a group, they were unable to play normally as the interface would not load on their

side within the allocated amount of time. Extending the allocated time beyond 80 seconds per decision and 20 seconds per review proved to not improve performance. As a result and to avoid loosing too many groups, we decided to introduce ‘ghost’ subjects, who could step in and take the place of those who dropped out. In practice this worked as follows.

We allocate subjects into groups of 6 rather than 5, and assign one subject to be a ‘ghost’. The main 5 subjects play the game just as described above. The experience for the ‘ghost’ is very similar. In every round of the game, the ‘ghost’ is randomly assigned to one position in the environment. Once everyone in the group makes their decisions, the outcome of the ghost is decided based on her actions and the actions of the subjects in the group who occupy the other four positions in the environment. This way, actions of the ‘ghost’ subject do not affect the outcomes of the group as long as no dropout occurs, but the ghost receives the experience of being part of that group. If a dropout occurs, the ‘ghost’ simply overtakes as part of the main group and the experiment proceeds as normal. The process is entirely seamless for all subjects. Consequently, we allow for up to 20% change in the group composition and preserve the group.

Of our 83 groups, 18 experienced a dropout overtaken by a ghost. 8 of those occurred in Baseline, 9 between Baseline and Intervention (i.e. due to a participant in the ‘main’ group failing the understanding quiz), and 1 in Intervention. In our main analysis, we consider data from 415 subjects including those 18 ‘ghosts’ but excluding data from the dropouts. As a robustness check, we check that the results are not sensitive to alternative specifications, including using data from dropouts before they were disqualified and excluding data from groups with dropouts altogether.

3 Dataset

Our dataset contains decision data of 415 participants. Each subject participated in exactly one session. For all treatments, we collected data for at least 20 groups of 5 participants each, and roughly half of the groups were from the Hubei province. In particular, we collected an 11th group from Hubei province with a fine intervention in the superspreader environment, and 2 further groups from the rest of China in the homogeneous environment with a fine intervention. In each treatment, participants interacted for 40 rounds (20 Baseline and 20 Intervention), to a total of $16,600$ decisions. We also match the experimental data with data from the recruitment survey.

Apart from data on subjects’ decisions in the experiment, we collect data on a set of variables, which can be broadly categorized as follows: demographics, preferences, and location-based controls. Table S1

presents summary statistics for some of these controls.

Table S1: Summary statistics the main controls.

Variable	$\bar{X}$	s.d.	Comments
Demographic controls			
Age	35.13	10.23	measured in years
Gender	0.47	0.50	female = 1
Education	18.7	1.48	measured in years
Employed	0.76	0.43	yes = 1
Religious	0.14	0.35	yes = 1
Preference controls			
BRET score	41.76	33.14	$\in [0, 100]$
SVO type	0.49	0.50	prosocial = 1
Residence controls			
Hubei	0.49	0.50	resides in Hubei = 1
Distance from Wuhan	4.92	4.50	in 100's of kilometers

Sample size is 414, because 1 subject did not complete post-experimental questionnaire and BRET; $\bar{X}$ – mean value, or proportion in case of binary variables; s.d. – standard deviation.

Demographic controls. All participants are resident in China, 47% are female, and the mean age is 35 years. An average subject in our sample has 18.7 years of education. Note that we estimate years of education using subjects' highest qualification reported. We assume that all subjects took the standard number of years to complete each qualification, and undertook no education that did not lead to a qualification. 76% of the sample are either employed or entrepreneurs. 14% are religious, with 7.5% and 1% respectively identifying Buddhism and Taoism as their religion, and 5.5% reporting to practice some other religion.

Preference controls. Further, as explained in the previous sections, we collect information on subjects' social value (SVO) and risk (BRET) preferences. The distributions of both for our sample are summarized in Figure S1. The average subject in the sample is moderately risk averse, with a BRET score of 42 boxes. When it comes to social values, as captured by the SVO, 51% of our subjects are classified as individualists. A further 49% are prosocials. We have 1 subject who classified as altruistic, and no subjects classified as competitive. For both BRET [6] and SVO [5], the resulting distributions are similar to the ones typically obtained in laboratory experiments.

Location-based controls. As part of recruitment, we collect data on participants province of residence,

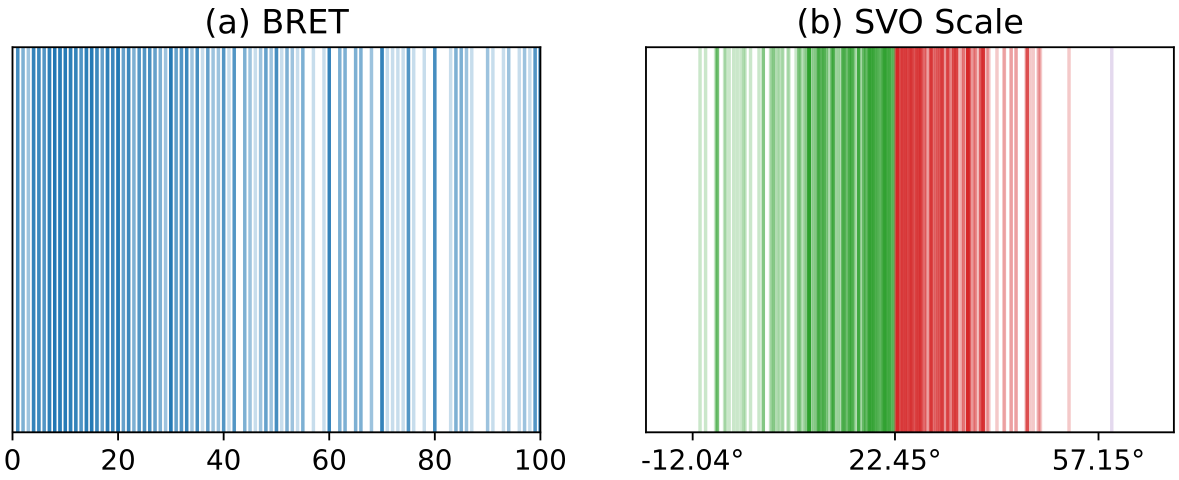

Figure S1: Bomb Risk Elicitation Task (BRET) and Social Value Orientation (SVO) Scale distributions for the sample. We draw a vertical line on the subplots for each subject whose score in BRET/SVO is of the corresponding value. More intense line color indicates that more subjects are concentrated at that value. For BRET, higher value corresponds to greater risk-seeking. For SVO the classification is as follows: angle $\geq$ 57.1° – altruist; $\geq$ 22.45° and $<$57.15° – prosocial; $<$22.45° and $\geq$ 12.04° – individualist; $<$-12.04° – competitive. Sample size is 414 (BRET), 415 (SVO).

while in the experiment we collect subjects' IP-addresses, which we then use to back out the province. Figure 1 (see main text) shows counts of subjects per province (based on data from recruitment). The province recorded from recruitment and IP from the experiment differs for 37 subjects in our sample. Note that this is not surprising, since, while IP data is 99% accurate for country identification, it is only 50-80% accurate for cities and regions. Further, the survey company responsible for recruitment has confirmed that some of those subjects were on a business trip when participating in the experiment, while others were using VPN. Excluding those subjects does not alter the distribution substantially.

4 Methodology: data analysis

4.1 Convergence

Figure S2 presents the evolution of average propensity to practice distancing at the individual level, separately for Hubei province and the rest of China, in both Baseline and Intervention (separately for fine and nudge).

Experiments on public good games typically exhibit a decline in contributions to the public good for several rounds of the experiment before contributions converge to a stable level [10]. Similarly, in our experiment, subjects exhibit a similar pattern in both Baseline and Intervention as shown in Figure S2.

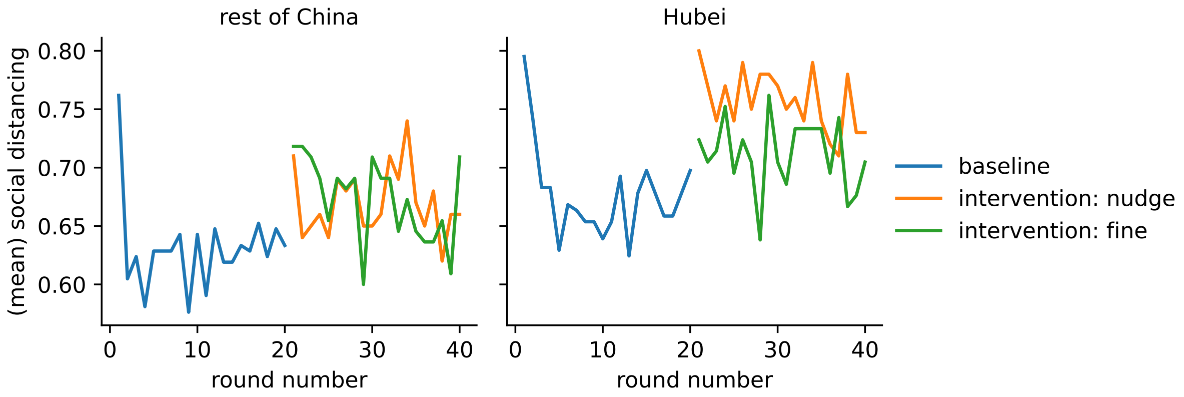

Figure S2: Mean distancing levels in Baseline and Intervention, separately for Hubei province and the rest of China.

Since we are interested in the limiting outcomes of this convergence behavior, the first step of our analysis is to determine the cut-off round, after which the majority of subjects converge to a stable strategy. We define individual convergence as follows:

Definition 1 *A participant converges to a strategy s by round n if (i) she used this strategy for the last k rounds (including n), and (ii) in all subsequent rounds $[n+1, 20]$ the number of consecutive deviations from the chosen strategy does not exceed a.*

We consider three types of convergence strategies. In both homogeneous and superspreader environments, we look at the strategy where the subject always chooses the same action. For the superspreader environment, we also consider two extra strategies. In one strategy the participant always chooses the same action when she is in the superspreader position and the complement action when she is a recipient. In the other strategy, she always chooses the same action when she is the superspreader and alternates between the two actions when a recipient.

We set $k = 4$ and $a = 2$. Consequently, to be considered converged at a round, a subject must choose the same strategy for the last four rounds, and not deviate from that strategy for more that two consecutive rounds. Moreover, the earliest a subject can be considered to converge to a stable strategy is by round 4.

Table S2 summarizes our convergence analysis using the above definition. From the table, we can see that by round 11 at least 77% of subjects in Baseline and 86.3% in Intervention converge to a stable strategy. In fact, when disaggregated by treatment, the lowest and highest convergence rates are 70.7% (star in Baseline) and 87.4% (fine in Intervention). Further analysis in Section 6 shows that our convergence analysis is robust top using a more/less conservative definition of convergence.

Table S2: Individual convergence analysis.

Network	Type of intervention	Population	n	$> 70\%$ converged by round ...	...% converged by round 11
**Baseline**					
all	all	all	415	8	77.8
complete	all	all	210	7	83.8
star	all	all	205	10	70.7
all	all	Hubei	205	8	76.6
all	all	non-Hubei	210	9	79.0
**Intervention**					
all	all	all	415	6	86.3
complete	all	all	210	5	86.7
star	all	all	205	6	85.9
all	all	Hubei	205	6	73.2
all	all	non-Hubei	210	6	83.8
all	fine	all	215	5	87.4
all	nudge	all	200	6	85

4.2 Parametric analysis

To analyze determinants of social distancing behavior in our experiment, we employ the following econometric framework. We assume that individual social distancing decision – a binary variable – is a linear function of our treatment variables and a range of controls. Consequently, we have the following linear probability model (LPM):

$$y_{it} = x_{it}\beta + v_i + \epsilon_{it}, \tag{3}$$

where

$$\Pr(y_{it} = 1|x_{it}) = x_{it}\beta + v_i. \tag{4}$$

The model assumes that the subject-specific random effect v_i is normally distributed, and error term ϵ_{it} is normally distributed and clustered at the group level.

The choice of a LPM over non-linear models is motivated by easy interpretation of coefficients, especially when it comes to interaction effects. However, as shown in Section 6, our results are not sensitive to a choice of model, as the estimated average marginal effects for the random effects Logit and Probit models are very similar.

Since the majority of subjects in our dataset exhibit clear convergence in behavior, our econometric analysis utilizes data from rounds 11-20 of both Baseline and Intervention – i.e. once participants behavior has converged to a stable strategy. In Section 6 we show that our key results are robust to using all of the data.

We perform econometric analysis in several stages, by consecutively adding controls which may plausibly explain social distancing decisions. To keep this section concise, full details of all our specifications are in Section 5.

5 Results

5.1 Overall effects

In our shortest specification (F1 of Table S3) we add dummies for experimental treatments: fine and nudge interventions, and the superspreader environment. As we randomly assign participants to these treatments, we are confident that their effects are causal.

Next, we add controls for social demographics and risk preferences and social preferences (F2), and a variable that captures the distance of one's place of residence from Wuhan in 100's of kilometers (F3).

The next specification (F4) is an auxilary one. Here, we replace distance from Wuhan in M1 with a dummy variable equal to one if the participant is from Hubei province and zero otherwise.

Further, using the data from the Oxford COVID-19 Government Response Tracker (OxCGRT), we construct an index of average overall government response for the period of 23rd Jan-2nd May 2020 [11]. This results in a single value of an index for each of the provinces in our sample. The correlation between distance from Wuhan and this index for the 20 cities in our sample is -0.6683 (t-test, $p = 0.0001$). Our next specification (F5) replaces distance from Wuhan with this index. Note that specifications F3-F5 are the full versions of the reduced M1-M3 reported in the main text.

Finally, to better understand the impact of the superspreader environment, in F6 we replace the superspreader environment dummy with two dummies – one for being in the superspreader position and another one for being the recipient.

Below we briefly summarize the main results of this exercise. We refer to effects that are significant at 1% level, as being very significant, at 5% level – as significant, and 10% level – as marginally significant.

Interventions. Fine for not practicing social distancing appears to work in China, but its overall effect

Table S3: Main regression results

Dependent variable:	social distancing (1 = yes)					
**Model**[a]	F1	F2	F3	F4	F5	F6
**Independent variables:**						
Fine treatment	0.0322*	0.0341*	0.0341*	0.0343*	0.0343*	0.0343*
	(0.0192)	(0.0196)	(0.0195)	(0.0195)	(0.0195)	(0.0195)
Nudge treatment	0.0608***	0.0606***	0.0605***	0.0603***	0.0603***	0.0603***
	(0.0148)	(0.0148)	(0.0148)	(0.0148)	(0.0148)	(0.0149)
Superspreader environment	-0.0406	-0.0440	-0.0491	-0.0494	-0.0481	
	(0.0322)	(0.0323)	(0.0314)	(0.0312)	(0.0311)	
Superspreader						0.0718**
						(0.0317)
Recipient						-0.0796**
						(0.0328)
Gender (1 = female)		0.0126	0.0110	0.0060	0.0086	0.0120
		(0.0312)	(0.0311)	(0.0312)	(0.0311)	(0.0310)
Age		0.0008	0.0014	0.0018	0.0019	0.0014
		(0.0016)	(0.0017)	(0.0017)	(0.0018)	(0.0017)
Years of education		0.0233**	0.0241**	0.0243**	0.0241**	0.0246**
		(0.0120)	(0.0119)	(0.0116)	(0.0117)	(0.0119)
Employed or entrepreneur (1 = yes)		0.0920**	0.1040**	0.1050**	0.1030**	0.1030**
		(0.0435)	(0.0430)	(0.0430)	(0.0427)	(0.0431)
Religious (1 = yes)		0.0328	0.0344	0.0349	0.0308	0.0343
		(0.0473)	(0.0479)	(0.0476)	(0.0471)	(0.0479)
Risk score		-0.0003	-0.0004	-0.0004	-0.0005	-0.0004
		(0.0005)	(0.0005)	(0.0005)	(0.0005)	(0.0005)
Prosocial values (1 = yes)		0.0295	0.0287	0.0286	0.0302	0.0279
		(0.0293)	(0.0289)	(0.0287)	(0.0287)	(0.0290)
Distance from Wuhan (100's km)			-0.0070**			-0.0071**
			(0.0031)			(0.0031)
Hubei residence (1 = yes)				0.0852***		
				(0.0311)		
OxCGRT index					0.0076**	
					(0.0034)	
Constant	0.670***	0.196	0.192	0.101	-0.389	0.184
	(0.0208)	(0.2080)	(0.2080)	(0.2110)	(0.3660)	(0.2080)
No of observations:	8,300	8,280	8,280	8,280	8,280	8,280
No of subjects[b]:	415	414	414	414	414	414

Notes: Standard errors (reported in parentheses) are clustered at the group level. *** $p < 0.01$, ** $p < 0.05$, * $p < 0.1$. **(a)** All regressions use Linear Probability Model. **(b)** One subject did not complete the post experimental questionnaire and BRET.

in the experiment is about 3.4 percentage points and is marginally significant in all models. Conversely, the effect of the nudge is almost twice in magnitude (6.0 percentage points) and is very significant in all specifications.

Environment. Overall, the effect of the superspreader environment is not significant in F1-F5. However, unpacking the superspreader environment into superspreaders and recipients (F6), we can see that superspreaders practice 7.2 percentage points more social distancing than subjects in the homogeneous setting and the effect is significant. Conversely, recipients practice 8.0 percentage points less distancing

than subjects in the homogeneous setting, again, with the effect being significant.

Demographics. According to Table S3, an extra year of education is associated with 2.4 percentage points more social distancing. Further, employed subjects or those who run their own business, practice 10.4 percentage points more social distancing. Both effects are significant.

Preferences. Overall, we do not find evidence that subjects' risk preferences and social values affect their social distancing decisions.

Distance from Wuhan. We find that subjects who reside further away from Wuhan practice less social distancing in the experiment (F3). The estimated size of the effect is 7.0 percentage points for 1,000 km and is significant. We also find that residing in Hubei province results in 8.5 percentage points higher propensity to practice social distancing compared to the rest of China (F4).

Strictness of government response. Harsher COVID-19 related measures undertaken by the government during the initial lockdown are associated with more social distancing. Specifically, an extra 10 points on the average OxCGRT index contributes an increase of 7.6 percentage points in the individual propensity to social distance (F5).

5.2 Heterogeneities

To better understand the driving forces behind the differences in the observed propensity to social distancing in Hubei province and outside of it, we perform the following analysis. We interact every variable in specification Table S3 F1 with the Hubei dummy and calculate the average marginal effects of each variable separately for Hubei and the rest of China. The resulting output is in Table S4.

Interventions. We find that fine for not practicing social distancing is effective in Hubei province but not outside of it. On the other hand, the nudge appears to be effective throughout China.

Environment. Theory predicts that there should be less social distancing in the superspreader environment, trivially due to the network of interactions being less dense. Subjects from Hubei province are, however, not responsive to the superspreader environment – the amount of social distancing is not significantly different from that observed in the homogeneous environment. Outside of Hubei, however,

subjects do less social distancing in the superspreader environment, and the effect is significant.

Demographics. Outside of Hubei province, more years of education is associated with more social distancing, and the effect is very significant. The same effect is not observed in Hubei province. On the other hand, subjects from Hubei who were employed or had their own business during the experiment, do significantly more social distancing, but the effect is not observed in the rest of China.

Preferences. We find that risk-seeking is associated with less social distancing outside of Hubei province with the effect being significant. Within Hubei province, the effect is not observable. Further, having prosocial values increases individual propensity to practice social distancing for subjects from the Hubei province but not the rest of China, with the effect being significant.

Table S4: Average marginal effects separately for the rest of China and Hubei province

**Dependent variable:**	social distancing (1 = yes)			
	rest of China		Hubei province	
**Independent variables:**				
Fine treatment	0.0090	(0.0248)	0.0602**	(0.0290)
Nudge treatment	0.0671***	(0.0189)	0.0544**	(0.0226)
Superspreader environment	-0.1109**	(0.0459)	-0.0184	(0.0393)
Gender (1 = female)	-0.0578	(0.0416)	0.0593	(0.0466)
Age	0.0020	(0.0021)	0.0008	(0.0027)
Years of education	0.0459***	(0.0137)	-0.0041	(0.0168)
Employed or entrepreneur (1 = yes)	0.0450	(0.0719)	0.1313**	(0.0582)
Religious (1 = yes)	0.0919	(0.0598)	-0.0055	(0.0682)
Risk score	-0.0015**	(0.0007)	0.0003	(0.0007)
Prosocial values (1 = yes)	0.0087	(0.0390)	0.0775*	(0.0431)
No of observations:	8,280			
No of subjects:	414			

Notes: Standard errors (reported in parentheses) are clustered at the group level. *** $p < 0.01$, ** $p < 0.05$, * $p < 0.1$. The regression is a Linear Probability Model. One subject did not complete the post experimental questionnaire and BRET.

6 Robustness checks

This section contains a selection of robustness checks. Section 6.1 reports robustness checks for our main results, while Section 6.2 contains further checks for our convergence analysis.

6.1 Main results

This section present 6 robustness checks on our main results, presented in Table S5. Each is discussed in turn.

In R1 of Table S5 we re-estimate F4 from Table S3 using all data. From the table we can see that none of the estimates of the key parameters of interest change materially. Re-estimating any other model from Table S3 using all data results in the qualitatively similar observation. This suggests that our results are not sensitive to discarding data pre-convergence.

Next, R2 reports marginal effects of each of the variables from specification in F4 using a logistic regression in place of a linear probability model. Similarly, R3 reports marginal effects from a probit regression applied to the same specification. As we can see, the point estimates of all parameters remain practically unchanged. These specifications suggests that our results are not driven by the choice of model.

Recall that 18 of our groups experienced one subject dropping out midway through the experiment. As explained in Section 2, we replaced these dropouts by a 'ghost' subject. In R4 we re-estimate F4 but discard data from all groups where a 'ghost' was activated due to a subject dropping out. Again, none of the estimates change materially, meaning that our experimental procedure of replacing dropouts does not drive our results.

Next, in our main analysis we use self-reported residence data to determine subjects' place of residence. It is possible, that this data is not very reliable. To investigate this possibility, we re-estimate specification in F3 now using location data as determined by subjects' IP-addresses when completing the experiment. Note that for 4 subjects in our sample the IP-address identified country as being other than China (e.g. Singapore of Thailand). Based on the information that we have, these subjects were using VPN when completing the study. Consequently, we exclude them from the analysis when estimating R5. Again, the obtained coefficients are not materially different from those in F3.

Finally, when looking at the association between OxCGRT index and social distancing behavior in the experiment we use data for the period 23rd Jan 2020 - 02nd May 2020. As a robustness check, in R6 we estimate the same index for the period 23rd Jan 2020 - 02nd May 2021 and use it as a control. This index is completely insignificant.

Table S5: Main regression robustness checks

**Dependent variable:**	social distancing (1 = yes)					
**Model[a]:**	R1	R2	R3	R4	R5	R6
**Independent variables:**						
Fine treatment	0.0344**	0.0343*	0.0324*	0.0452**	0.0339*	0.0340*
	(0.0158)	(0.0193)	(0.0189)	(0.0218)	(0.0196)	(0.0196)
Nudge treatment	0.0658***	0.0546***	0.0533***	0.0729***	0.0547***	0.0606***
	(0.0134)	(0.0136)	(0.0137)	(0.0172)	(0.0152)	(0.0148)
Superspreader environment	-0.0493*	-0.0455	-0.0450	-0.0432	-0.0568*	-0.0437
	(0.0293)	(0.0298)	(0.0307)	(0.0353)	(0.0315)	(0.0323)
Gender (1 = female)	-0.00103	-0.00730	-0.00668	-0.00108	0.0134	0.00975
	(0.0295)	(0.0305)	(0.0315)	(0.0371)	(0.0309)	(0.0312)
Age	0.00147	0.00197	0.00201	0.00147	0.00130	0.000791
	(0.00159)	(0.00161)	(0.00165)	(0.00190)	(0.00169)	(0.00159)
Years of education	0.0249**	0.0217*	0.0212*	0.0154	0.0247**	0.0231*
	(0.0107)	(0.0112)	(0.0118)	(0.0120)	(0.0120)	(0.0119)
Employed or entrepreneur (1 = yes)	0.0995***	0.0970**	0.0957**	0.133***	0.107**	0.0925**
	(0.0386)	(0.0400)	(0.0412)	(0.0459)	(0.0428)	(0.0436)
Religious (1 = yes)	0.0384	0.0347	0.0337	0.0849*	0.0430	0.0345
	(0.0446)	(0.0474)	(0.0490)	(0.0491)	(0.0477)	(0.0476)
Risk score	-0.000314	-0.000502	-0.000501	-0.000387	-0.000508	-0.000324
	(0.000478)	(0.000497)	(0.000514)	(0.000560)	(0.000500)	(0.000513)
Prosocial values (1 = yes)	0.0281	0.0336	0.0341	0.0292	0.0340	0.0290
	(0.0269)	(0.0270)	(0.0279)	(0.0317)	(0.0290)	(0.0293)
Hubei residence (1 = yes)	0.0854***	0.0789***	0.0783***	0.0680*		
	(0.0285)	(0.0293)	(0.0302)	(0.0352)		
Distance from Wuhan (100's km)					-0.00675**	
					(0.00322)	
OxCGRT index for 2021						-0.00283
						(0.00362)
Constant	0.109	-	-	0.243	0.187	0.349
	(0.196)	-	-	(0.226)	(0.209)	(0.300)
No of observations:	16,560	8,280	8,280	6,480	8,200	8,280
No of subjects[b]:	414	414	414	324	410	414

Notes: Standard errors (reported in parentheses) are clustered at the group level. *** $p < 0.01$, ** $p < 0.05$, * $p < 0.1$. **(a)** R2 is a Logistic regression and R3 is a probit regression. Other models use a Linear Probability Model. **(b)** One subject did not complete the post experimental questionnaire and BRET.

6.2 Convergence

Recall that in Section 4.1 we define a subject to be converged to a stable strategy by a particular round if she followed this strategy for the last four rounds and in subsequent rounds does not deviate from this strategy for more than two consecutive rounds. As a robustness check, we consider allowing for one and three consecutive deviations respectively. Figure S3 plots the share of converged participants for each round separately by parts when allowing for 1-3 consecutive deviations ($a \in [1, 3]$). We can see that the share of converged subjects does not change much when we allow for a more/less conservative definition. In particular, with $a = 1$ the share of subjects who converge by round 11 in Baseline drops to 72.7% while in Intervention it reaches 81.2%. With $a = 3$ the share of subjects who converge by round 11 in Baseline and Intervention stands at 83.4% and 87.5% respectively.

As an extra robustness check for our convergence analysis, we recalculate convergence statistics for our sample, excluding the data from our 18 ‘ghost’ subjects. The results are summarized in Table S6. We can see, that, with ‘ghosts’ excluded, the share of converged subjects rises both in Baseline (from 77.8% to 87.4%) and Intervention (from 86.3% to 89.9%).

The above analysis suggests that it is reasonable to claim that the absolute majority of subjects converge to a particular strategy by round 11 in both parts of the experiment.

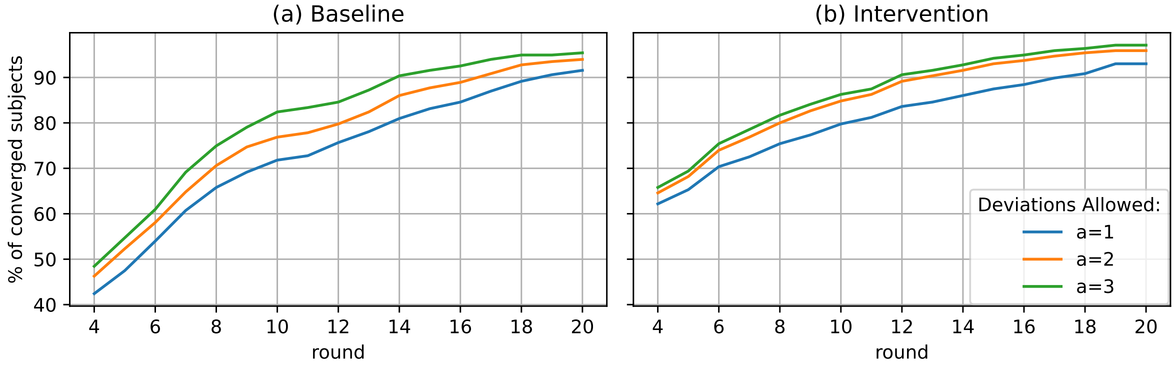

Figure S3: Evolution of the share of converged participants throughout the experiment separately for Baseline and Intervention, $a \in [1, 3]$. Note: we exclude the first 3 rounds in both parts since $k = 4$.

Table S6: Individual convergence analysis: robustness check

Network	Type of intervention	Population	n	$>70\%$ converged by round ...	...% converged by round 11
**Baseline**					
all	all	all	397	7	87.4
complete	all	all	202	7	83.7
star	all	all	195	6	91.3
all	all	Hubei	198	7	86.4
all	all	non-Hubei	199	7	88.4
**Intervention**					
all	all	all	397	5	89.9
complete	all	all	202	5	87.6
star	all	all	195	5	92.3
all	all	Hubei	198	5	90.4
all	all	non-Hubei	199	5	89.4
all	fine	all	207	4	90.3
all	nudge	all	190	5	89.5

References

[1] A. Mas-Colell, M. D. Whinston, J. R. Green, *et al.*, *Microeconomic theory*. Oxford University Press, 1995.

[2] D. Acemoglu, S. Johnson, and J. A. Robinson, "Institutions as a fundamental cause of long-run growth," *Handbook of Economic Growth*, vol. 1, pp. 385–472, 2005.

[3] R. D. Putnam, R. Leonardi, and R. Nanetti, *Making Democracy Work: Civic Traditions in Modern Italy*. Princeton: Princeton University Press, 1993.

[4] E. U. Weber, A.-R. Blais, and N. E. Betz, "A domain-specific risk-attitude scale: measuring risk perceptions and risk behaviors," *Journal of Behavioral Decision Making*, vol. 15, no. 4, pp. 263–290, 2002.

[5] R. O. Murphy, K. A. Ackermann, and M. Handgraaf, "Measuring social value orientation," *Judgment and Decision making*, vol. 6, no. 8, pp. 771–781, 2011.

[6] P. Crosetto and A. Filippin, "The "bomb" risk elicitation task," *Journal of Risk and Uncertainty*, vol. 47, no. 1, pp. 31–65, 2013.

[7] G. Charness, U. Gneezy, and B. Halladay, "Experimental methods: Pay one or pay all," *Journal of Economic Behavior and Organization*, vol. 131, pp. 141 – 150, 2016.

[8] D. L. Chen, M. Schonger, and C. Wickens, "oTree – an open-source platform for laboratory, online, and field experiments," *Journal of Behavioral and Experimental Finance*, vol. 9, pp. 88–97, 2016.

[9] F. Holzmeister and A. Pfurtscheller, "oTree: the "bomb" risk elicitation task," *Journal of Behavioral and Experimental Finance*, vol. 10, pp. 105–108, 2016.

[10] J. O. Ledyard, *Public goods: A survey of experimental research*. California Institute of Technology, 1994.

[11] D. J. Hunter, "COVID-19 and the stiff upper lip – The pandemic response in the United Kingdom," *New England Journal of Medicine*, vol. 382, no. 16, p. e31, 2020.

7 Instructions: Translation to English

In this section we present translations of the original instructions into English. Section 7.1 presents instructions for the experiment. Analogously, Sections 7.2 and 7.2 present instructions for the social preferences (SVO) and risk preferences (BRET) elicitation tasks respectively. Since the experiment was conducted in China, all instructions were written in Mandarin. Section 7 contains English translations.

7.1 Main Experiment

This section contains instructions for Baseline and Intervention parts of the experiment. Note that the type of network and intervention do not feature in this part of instructions. For the Intervention (Part 2), we show instructions for the fine. Instructions for the nudge are similar, except that instead of explaining how the fine is implemented, participants are asked to watch a 3-minute video. The video can be obtained from the authors on request.

Part 1: Instructions (page 1/6)

Welcome to this interactive experiment!

This experiment consists of two parts. You will be paid a fixed reward of ¥5 for completing all parts of the experiment. Additionally, you can earn points for your choices in Parts 1 and 2, which will be converted into ¥at the end of this experiment. There may also be a Bonus Task at the end of the experiment.

During Parts 1 and 2 of the experiment, you and other recruited people are randomly assigned to form groups of five for an interactive mini-game. During this process, the identities of you and others are kept confidential, and the identities of others are not known.

The expected duration of the experiment is 30 minutes and your average expected total earnings will be ¥13-15 excluding the Bonus Task. Note that you can earn less or more than this amount depending on your choices and the choices of others in your group.

At the beginning of Part 1 of the experiment, you will be randomly allocated to a group of 5 and you will remain in this group for the duration of Parts 1 and 2 of the experiment. Note that you might have to wait while we are matching you with 4 other people, but we will compensate you for the time you wait.

Since this experiment is interactive, it is important that you remain continuously attentive, otherwise you may slow down others and may even be disqualified from the experiment.

The instructions on the next 5 pages explain the rules of the game. You will receive more information about Part 2 after you complete Part 1.

All participants are given the same instructions. It is important that you read these instructions carefully. Note that there is no deception in this experiment.

Once you read the instructions, you will be required to pass a short understanding Quiz. If you fail the Quiz, you will not be allowed to take part in the experiment and will not receive the fixed reward.

To continue to the instructions, press the 'Next' button below.

Part 1: Instructions (page 2/6)

In Part 1 of the experiment you are asked to play a game with the other members of your group. In what follows, you and the other members of your group are referred to as participants.

At the start of the game, you are presented with a diagram with 5 circles labeled by capital letters (P, E, C, M, Q) and lines between them.

Each circle represents a participant, and the circle and line segment mark his position in relation to other participants. At the start of the game, each participant is randomly allocated to one of these positions in the diagram. Your position is the one colored in blue.

The lines between positions indicate the structure of interactions between participants in these positions. These lines indicate which participants interact with one another in the game.

An example is the diagram below. Here, you are in position M and directly interact with participants in positions P and E, but you do not interact directly with C and Q.

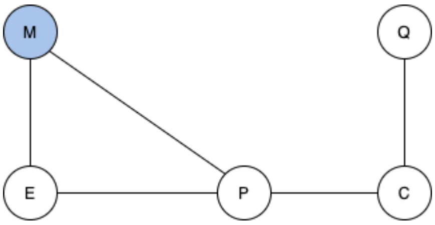

The next page of the instructions explains the choice you need to make in the game. To continue to the next page, press the 'Next' button below. To go to the previous page, press the 'Back' button.

Part 1: Instructions (page 3/6)

In the game, you and the other participants face a risk of getting infected with the new coronavirus (COVID-19).

The main symptoms of COVID-19 are shortness of breath, a high fever and a new, continuous cough. Most patients experience mild symptoms and recover in 1-2 weeks, but cases can progress to pneumonia and organ failure in the most vulnerable individuals.

After you learn your position in the diagram, your need to choose whether to practice social distancing. Below you can see what the buttons to make your choice look like.

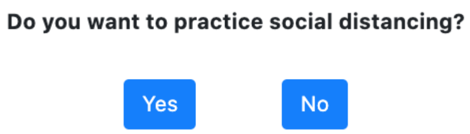

You have 80 seconds to make your choice and the timer is displayed at the top of the interface throughout the experiment. If you do not make a choice within the allowed time, your choice is automatically recorded as a ‘No’.

After everyone in your group has made their social distancing choice, the computer randomly chooses one and only one participant to contract the new coronavirus directly. If a participant does not practice social distancing and is randomly picked by the computer then s/he gets infected for sure. In other words, if you do not practice social distancing there is a 20% chance you get infected with the new coronavirus directly.

Social distancing gives you some level of protection against COVID-19. If you practice social distancing and you are the participant randomly chosen by the computer to be infected then the computer flips a fair coin. If the coin flip is Head you are infected with COVID-19. If the coin flip is Tail then you are not infected with COVID-19. In other words, if you practice social distancing there is a 10% chance you get infected with COVID-19 directly.

A participant who practices social distancing cannot pass COVID-19 to other participants and cannot be infected with COVID-19 by another participant.

On the other hand, an infected participant who does not practice social distancing may infect other participants through contagion. In particular, other participants who do not practice social distancing face a risk of getting infected because COVID-19 may spread through interactions between the participants.

The next page of the instructions explains how COVID-19 spreads through interactions between participants. To continue to the next page, press the ’Next’ button below. To go to the previous page, press the ’Back’ button.

Part 1: Instructions (page 4/6)

A healthy participant who does not practice social distancing may get infected with COVID-19 through contagion by interacting with infected participants who do not practice social distancing. The probability a participant contracts COVID-19 through interaction with another participant is referred to as the rate of contagiousness of COVID-19.

Throughout Part 1 of the experiment the rate of contagiousness of COVID-19 is fixed at 65%.

Consider again the example diagram of interactions and, as an example, suppose that:

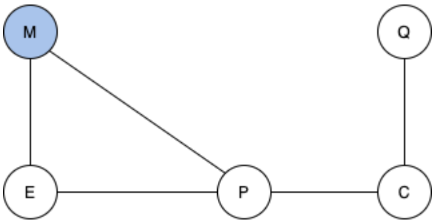

- You (participant M) do not practice social distancing.
- Participants in position E and Q practice social distancing, while participants in positions P and C do not.

In this example, suppose C is randomly picked by the computer to contract COVID-19 directly.

First, C has chosen not to practice social distancing so s/he gets infected for sure. Moreover, C can pass COVID-19 to other participants because s/he does not practice social distancing.

Second, E and Q cannot get infected with COVID-19 through contagion because they practice social distancing.

Next, P may get infected through contagion because s/he does not practice social distancing and interacts with C. This can happen with probability 65% – the rate of contagiousness.

Finally, you may also become infected by contagion through your interaction with P. Specifically, there is a 65% probability you might get infected through your interaction with P if s/he becomes infected. However, if P remains healthy you will also remain healthy for the duration of the game.

It follows that in this example if 1) you do not practice social distancing, 2) E and Q practice social distancing whereas P and C do not, and 3) C contracts COVID-19 directly, then you may become infected with probability 42.25%. The diagram below shows how this percentage is computed.

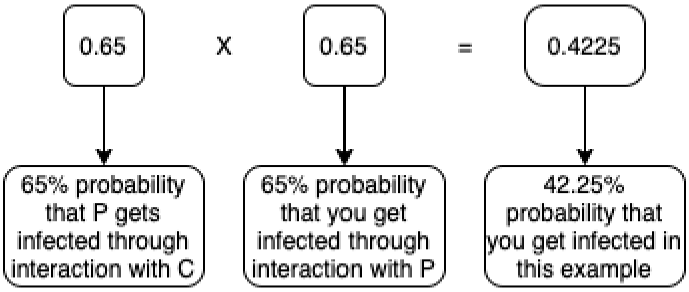

Note that you will not be informed of the choices of other participants at any point during the experiment.

The next page of the instructions explains how you earn points in Part 1 of the experiment. To continue to the next page, press the 'Next' button below. To go to the previous page, press the 'Back' button.

Part 1: Instructions (page 5/6)

At the end of the game you earn the following points depending on your social distancing choice and infection status:

- 100 points: if you did not practice social distancing and did not get infected;
- 65 points: if you practiced social distancing and did not get infected (100 points for being healthy minus 35 points cost of social distancing);
- 0 points: if you did not practice social distancing and got infected;
- -35 points: if you practiced social distancing and got infected (0 points for being infected minus 35 points cost of social distancing).

Note that if you fail to submit your social distancing choice then you will receive a penalty of 50 points.

The information about the points you can earn, the rate of contagiousness of COVID-19, the structure of interactions between participants and your position are always displayed on the screen when you make your social distancing choice.

Below you can see an example of how this part of the interface looks like. The diagram of interactions is on the right, while the textual information on the left reminds you of the rate of contagiousness and the possible outcomes.

Rate of contagiousness	65%
Points:	
- not practiced distancing, healthy	100
- practiced distancing, healthy	65
- not practiced distancing, infected	0
- practiced distancing, infected	-35

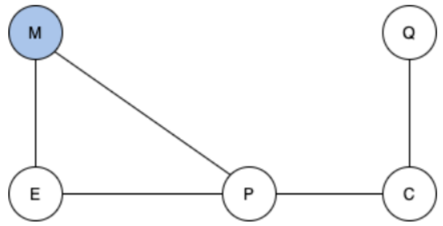

At the end of the game, you are reminded of the structure of interactions between participants, your position within that structure, and your social distancing choice. You are also informed of your infection status and the number of points earned.

Below you can see an example of how this part of the interface looks like.

Your distancing choice	practiced distancing
Your infection status	healthy
Points earned	65

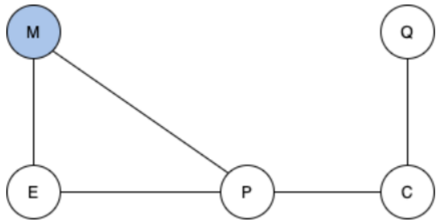

You have 20 seconds to review this information and the timer is always displayed at the top of the interface.

The next page of the instructions explains how points are converted into your earnings in ¥. To continue to the next page, press the 'Next' button below. To go to the previous page, press the 'Back' button.

Part 1: Instructions (page 6/6)

Part 1 of the experiment has 20 separate games as described in these instructions. The choice you make in one game has no effect on other games.

The only variation between games is the random reassignment of the positions of all the participants (including you) in the diagram of interactions. The participants assigned to your group, the structure of interactions between positions, the probability of contracting COVID-19 directly, the rate of contagiousness of COVID-19 and the number of points earned depending on social distancing choice and infection status remain unchanged.

At the end of each game, you can review the history of your choices and outcomes for the last 5 games. The table below shows an example of how your history after 9 games might look like.

Your History

Game	Position	Distancing Choice	Infection Status	Points Earned
9	Q	practiced distancing	healthy	65
8	E	practiced distancing	healthy	65
7	P	not practiced distancing	healthy	100
6	M	practiced distancing	infected	-35
5	C	not practiced distancing	infected	0

It is important that you make a choice in every game. If you fail to make a choice for 3 consecutive games, you will be disqualified from the experiment. In this case, you will not receive any payment for this experiment.

At the end of this experiment, the computer randomly picks 4 out of 20 games to determine your earnings for Part 1 of the experiment.

The points are converted to ¥at a rate of 50 points per ¥1.

Suppose that you earn 260 points in the 4 randomly drawn games. Then, your total earnings for Part 1 are ¥5.2.

To continue to the short Quiz, press the 'Next' button below. To go to the previous page of the instructions, press the 'Back' button.

Part 2: Instructions

You have completed Part 1 of the experiment and will now proceed to Part 2.

Below are the instructions for Part 2 of the experiment. It is important that you read these instructions carefully.

This part of the experiment also has 20 games, and you are assigned to the same group of 5 people as in Part 1. The structure of interactions between participants, the probability of contracting COVID-19 directly and the rate of contagiousness of COVID-19 are the same as in Part 1.

The single difference from Part 1 is that in Part 2 of the experiment you will receive a fine of 15 points in any game in which you do not practice social distancing.

Hence, in Part 2 of the experiment the points you earn at the end of the game are:

- 85 points: if you did not practice social distancing and did not get infected (100 points for being healthy minus 15 points fine);
- 65 points: if you practiced social distancing and did not get infected (100 points for being healthy minus 35 points cost of social distancing);
- -15 points: if you did not practice social distancing and got infected (0 points for being infected minus 15 points fine);
- -35 points: if you practiced social distancing and got infected (0 points for being infected minus 35 points cost of social distancing).

Note that if you fail to submit your social distancing choice then you will receive a penalty of 50 points.

Your earnings for Part 2 are computed in the same way as in Part 1. At the end of this experiment, the computer randomly picks 4 out of 20 games to determine your earnings for Part 2 of the experiment.

As in Part 1, the points are converted to ¥at a rate of 50 points per ¥1.

Suppose that you earn 300 points in the 4 randomly drawn games. Then, your total earnings for Part 2 are ¥6.

Before you can start Part 2 of the experiment, you must answer a Quiz question on the instructions above. If you fail to answer the question correctly, you will not be able to continue to Part 2 of the experiment. To continue to the short Quiz, press the 'Next' button below.

7.2 Social Value Orientation (SVO) Slider Measure

This section contains instructions to the Social value Orientation (SVO) task which participants complete as part of the recruitment survey.

Bonus Task: Instructions

You have answered the 2 qualifying questions correctly and are now in the Bonus Task.

In this Bonus Task you will be making a series of decisions about allocating money between you and another anonymous participant. All of your decisions will be completely confidential.

There is a total of 6 decisions to make which are independent of each other. For each decision, you are asked to pick the distribution of money between yourself and another anonymous participant that you

prefer, all values are stated in ¥. After you have made your decision, select the resulting distribution of money by clicking on the button below your choice. As you will see, your choices will influence both the amount of money you receive as well as the amount of money the other anonymous participant receives.

There are no right or wrong answers, this is all about personal preferences.

Every time 50 people complete the task, we will randomly pick two of them and pay them for the Bonus Task as follows. We will pick one of the 6 decisions, and randomly implement the decision of one of the two chosen participants.

For example, suppose we randomly chose persons X and Y out of those 50 participants, and that we further randomly chose to implement decision 3 of person Y. Suppose that in decision 3 Turker Y allocated ¥8.5 to themselves, and ¥6.5 to another anonymous participant. Therefore, persons X and Y will be paid ¥6.5 ¥8.5 respectively.

7.3 Bomb Risk Elicitation Task (BRET)

This section contains instructions to the Bomb Risk Elicitation Task (BRET) which participants complete after the main experiment.

Bonus Task: Instructions

Thank you very much for taking part in the experiment!

You are now in the Bonus Task in which you have an opportunity to earn an extra payment.

On the next page, you will see 100 boxes. As soon as you start the task by pressing the 'Start' button, one box is collected per second starting from the top left corner. Once collected, the box is marked by a tick symbol.

For each box collected, you earn ¥0.1.

One of the 100 boxes contains a bomb that destroys all of your earnings. You do not know where the bomb is located. You only know that the bomb can be in any box with equal probability.

Your task is to choose when to stop collecting the boxes and open those you have collected. You stop collecting boxes by pressing 'Stop' at any time. After that you open the boxes you have collected by pressing the 'Open' button. Note that once you press 'Stop' you cannot restart collecting boxes.

A dollar or a bomb symbol will be shown on each of the boxes you have collected.

If the bomb symbol does not appear, that means that you have not collected the box with the bomb. In this case, you earn the amount accumulated by the boxes you have collected.

If the bomb symbol appears, that means that you have collected the box with the bomb. In this case, you earn zero for the Bonus Task.

To proceed to the Bonus Task, press the button below.
